# Cytogenetic characterization and B chromosome diversity in direct-developing frogs of the genus *Oreobates* (Brachycephaloidea, Craugastoridae)

**DOI:** 10.3897/CompCytogen.v10i1.5718

**Published:** 2016-03-21

**Authors:** Juan Martín Ferro, Alberto Taffarel, Darío Cardozo, Jimena Grosso, María Pía Puig, Pablo Suárez, Mauricio Sebastián Akmentins, Diego Baldo

**Affiliations:** 1 Laboratorio de Genética Evolutiva, Instituto de Biología Subtropical (CONICET-UNaM), Facultad de Ciencias Exactas Químicas y Naturales, Universidad Nacional de Misiones; Félix de Azara 1552, CPA N3300LQF Posadas, Argentina; 2 Fundación Miguel Lillo, Instituto de Herpetología; Miguel Lillo 251, CP 4000, San Miguel de Tucumán, Tucumán; 3 Universidad Nacional de Salta (UNSa), Avenida Bolivia 5150, Salta, Argentina; 4 Laboratório de Citogenética, Instituto de Ciências Biológicas, Universidade Federal do Pará,Tv. Augusto Correia 1, CEP 66075-900, Belém, Pará, Brazil; 5 Centro de Investigaciones y Transferencia de Jujuy (CIT-JUJUY), CONICET-UNJu, Av. Bolivia 1711 (4600), San Salvador de Jujuy, Argentina

**Keywords:** Cytogenetics, accessory elements, ribosomal DNA, Anura

## Abstract

*Oreobates* Jiménez de la Espada, 1872 is a large group of South American frogs with terrestrial reproduction and direct development, located in the superfamily Brachycephaloidea. About 260 brachycephaloidean species have been cytogenetically studied so far, at least with standard techniques. However, this information represents fewer than 17% species of the family Craugastoridae Hedges, Duellman & Heinicke, 2008, where the genus *Oreobates* is included. In the present work, using a diversity of standard and molecular techniques, we describe the karyotype of *Oreobates barituensis* Vaira & Ferrari, 2008, *Oreobates berdemenos* Pereyra, Cardozo, Baldo & Baldo, 2014 and *Oreobates discoidalis* (Peracca, 1895), from northwestern Argentina. The three species analyzed showed a diploid karyotype with 2n = 22 biarmed chromosomes, fundamental number (FN) = 44, nucleolus organizer regions (NORs) located pericentromerically on pair 7, and a centromeric and pericentromeric C-banding pattern. We observed variations in the chromosome number in *Oreobates barituensis* due the presence of two morphs of B chromosomes, one medium-sized telocentric (B_T_) and another subtelocentric and smaller (B_st_). Both B chromosomes are mitotically stable and were recorded in all somatic and germinal cells analyzed. The B_T_ chromosome occurred at a maximum of one per individual (2n = 22+B_T_), and the other one was observed single (2n = 22 + B_st_) or as a pair in two doses (2n = 22 + 2B_T_). We additionally observed other supernumerary chromosomes in the three species analyzed, all of them euchromatic, small, dot-shaped and with instability during mitoses, showing a frequency of occurrence below 50% in studied specimens. The occurrence of polymorphic and spontaneous chromosomal rearrangements and supernumerary chromosomes is a recurrent feature reported in frogs with terrestrial habits (Brachycephaloidea and Hemiphractidae Peters, 1862), which suggests that Brachycephaloidea may be a promising group for studying the origin and maintenance of B chromosomes in anurans.

## Introduction

Superfamily Brachycephaloidea includes a large group of frogs with terrestrial reproduction and direct development, with more than a thousand species assigned to three families: Brachycephalidae Günther, 1858, Craugastoridae, and Eleutherodactylidae Lutz, 1954 (Frost 2015). From a cytogenetic perspective, about 26% of brachycephaloidean species were studied at least with conventional staining techniques, including 13% of Brachycephalidae (7 spp.), 17% of Craugastoridae (128 spp.) and 57% of Eleutherodactylidae (120 spp.) (Campos and Kasahara 2006, Schmid et al. 2010, Díaz et al. 2012, Kaiser et al. 2015). Brachycephaloidea presents an important karyotypic diversity, with diploid numbers (2n) ranging from 16 to 38, an unusually high frequency of spontaneous and polymorphic Robertsonian rearrangements, B chromosomes, and the occurrence of spontaneous somatic supernumerary chromosomes which do not form polymorphisms (Schmid et al. 2010).

Within Craugastoridae (subfamily Holoadeninae Hedges, Duellman & Heinicke, 2008), *Oreobates* Jiménez de la Espada, 1872 is a South American genus with 23 species that occurs on the lower slopes of the Andes from the upper Amazon basin in southern Colombia to northern Argentina, reaching eastwards some areas in western Brazil (Frost 2015). In Argentina, the genus is represented by three species, *Oreobates berdemenos* Pereyra, Cardozo, Baldo & Baldo, 2014, which is phylogenetically related to *Oreobates lundbergi* and characterized by an incomplete discoidal fold (Pereyra et al. 2014); and the cryptic species, *Oreobates barituensis* Vaira & Ferrari, 2008 and *Oreobates discoidalis* (Peracca, 1895), recovered as sister species in the most inclusive phylogenetic analyses, both with a complete discoidal fold (Padial et al. 2014, Pereyra et al. 2014). When compared to other Brachycephaloidea genera, the cytogenetic information available for *Oreobates* is extremely scarce and only two species were studied to date, *Oreobates discoidalis* (Brum-Zorilla and Sáez 1968 [as *Eleutherodactylus*], Schmid et al. 2010), and *Oreobates crepitans* (Bokermann, 1965) (Siqueira et al. 2009, as [*Pristimantis*]). Both taxa share the same chromosome formulae (2n = 22, FN = 44) and similar chromosomal morphology. In the latter the heterochromatin pattern and nucleolus organizer regions were described by C- banding and silver staining (Ag-NORs) respectively, whereas the karyotype of *Oreobates discoidalis* was only studied with standard staining techniques (Giemsa).

B chromosomes (Bs) are dispensable extra chromosomes in the standard karyotype (As) present in many taxa, and usually lack phenotypic effects on their hosts. Their prevalence in animal populations is highly variable, being one of the main causes of chromosomal polymorphism in eukaryotes (Jones and Rees 1982, Beukeboom 1994, Camacho et al. 2000). Until now, the presence of B chromosomes in Brachycephaloidea has been described in three species, *Craugastor* sp., *Eleutherodactylus gundlachi* Schmidt, 1920 and *Oreobates discoidalis*. However, another sort of chromosomal variation due to supernumeraries is frequently observed. Although they are associated with spontaneous chromosomal aberrations, their main difference with Bs lies in the fact that they are not polymorphic (Schmid et al. 2010).

In order to complement the karyotypic information available for *Oreobates*, in the present work we studied three species (*Oreobates barituensis*, *Oreobates berdemenos*, and *Oreobates discoidalis*), from several localities in northwestern Argentina. Chromosome morphology, heterochromatin distribution and composition, and location of nucleolar organizer regions are described. We discuss and evaluate the apparent chromosomal homogeneity observed for the genus *Oreobates*, in contrast to the variability reported by the presence of supernumerary chromosomes.

## Material and methods

We studied 64 specimens of both sexes of *Oreobates barituensis* (N = 40), *Oreobates berdemenos* (N = 14), and *Oreobates discoidalis* (N = 10). Chromosome preparations were obtained from bone marrow, gut epithelium and testes in males (Schmid et al. 2010). Animals were euthanized with 5% lidocaine and fixed in 4% formalin. Vouchers were preserved in 70% ethanol and stored in the herpetological collections of Fundación Miguel Lillo, Tucumán, Argentina (FML, and provisional field numbers MSA), and Laboratorio de Genética Evolutiva, Instituto de Biología Subtropical, Posadas, Misiones, Argentina (LGE, and provisional field numbers MSA). The complete list of specimens analyzed, collection sites, sex, and voucher numbers are detailed in the Supplementary file 1 online. The diploid number (2n) and Fundamental Number (FN) were obtained after counting at least ten cells per specimen. Mitotic and meiotic preparations were stained with a phosphate-buffered Giemsa solution (10%). Heterochromatic regions were identified by C-banding (Sumner 1972). Silver-staining (Howell and Black 1980) and fluorescent in situ hybridization (FISH) with a ribosomal 18S biotinylated probe (Pinkel et al. 1986) were carried out to evidence active nucleolar organizer regions Ag-NORs and the presence of repetitive rDNA, respectively. To study the nucleotide composition of heterochromatin (prevalence of repetitive sequences AT and CG), we used the fluorochromes base specific DAPI (4’, 6-diamidino-2-phenylindole) and CMA_3_ (Chromomycin A_3_), following Schweizer (1976). Karyotypes were arranged in decreasing size, according the nomenclature of Green and Sessions (1991, 2007). The relative length and centromeric index (CI) of chromosomes were obtained from mitotic metaphases using the software Micromeasure 3.3 (Reeves and Tear 2000).

The advertisement call of *Oreobates discoidalis* remains unknown, and those described by Ferrari and Vaira (2008) and Akmentins (2011) correspond to *Oreobates berdemenos* (Pereyra et al. 2014). To avoid misidentification of specimens, collections of *Oreobates discoidalis* were made near the type locality (San Miguel de Tucumán, Argentina), whereas individuals from Jujuy and Salta Provinces were considered as *Oreobates barituensis*, based on their advertisement calls as described by Vaira and Ferrari (2008) and Akmentins (2011). Specimens of *Oreobates berdemenos* were collected at the type locality of the species (Abra Colorada, Jujuy province, Argentina), and Nogalar de los Toldos (Salta province, Argentina). The identity of *Oreobates berdemenos* specimens was confirmed by morphological and/or acoustic characters (Supplementary file 1 online).

## Results

**Figure 1.**
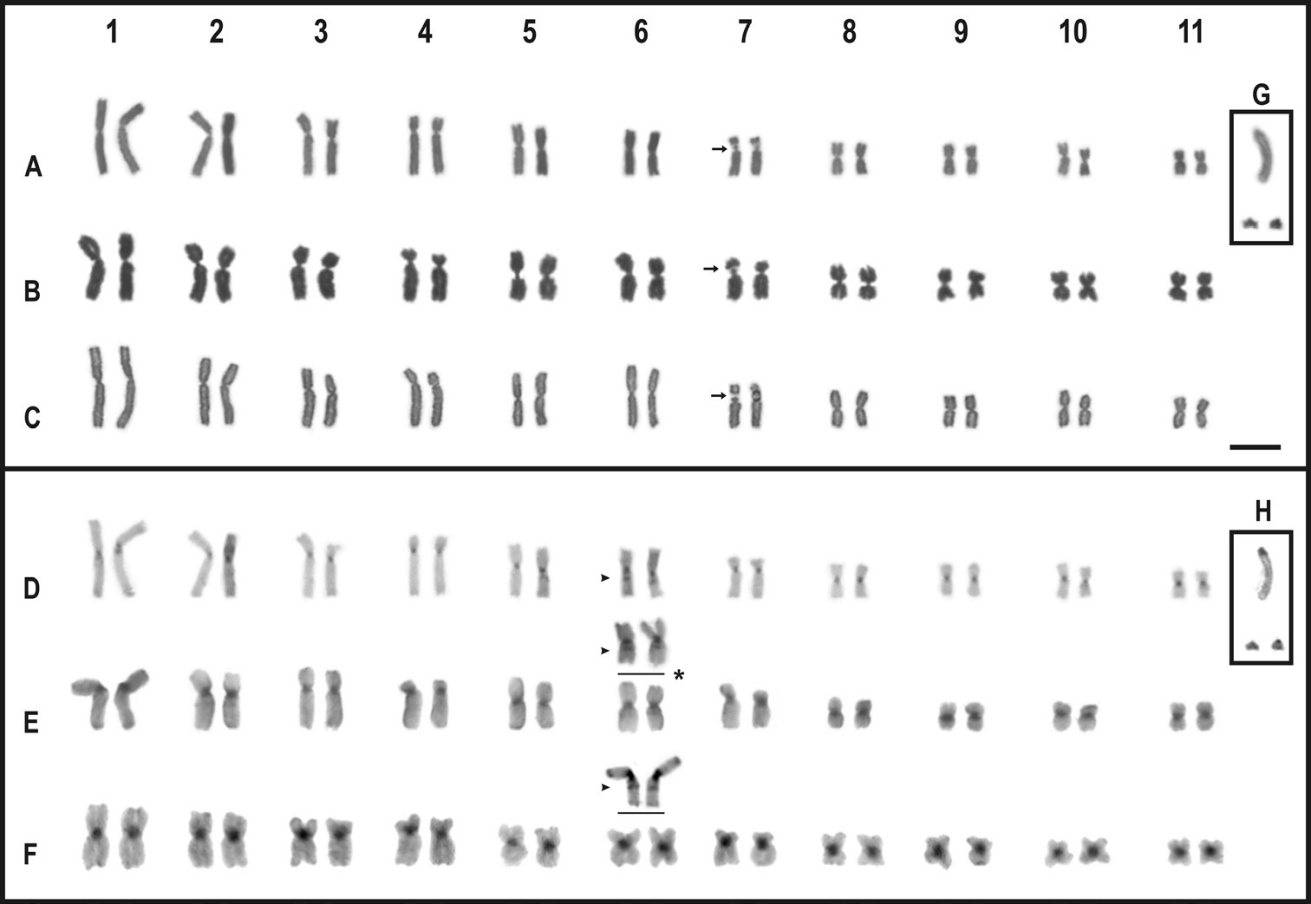
Karyotypes of *Oreobates barituensis* (**A, D**), *Oreobates berdemenos* (**B, E**), *Oreobates discoidalis* (**C, F**), the large telocentric B_T_ and the small subtelocentric B_st_ supernumerary chromosomes in *Oreobates barituensis* (Boxes **G** and **H**). Giemsa stained (**A–C, G**), C- banding (**D–F, H**). The insets (*) shows interstitial C-bands. Arrowheads point to C positive bands. Bar = 10 µm.

**Figure 2.**
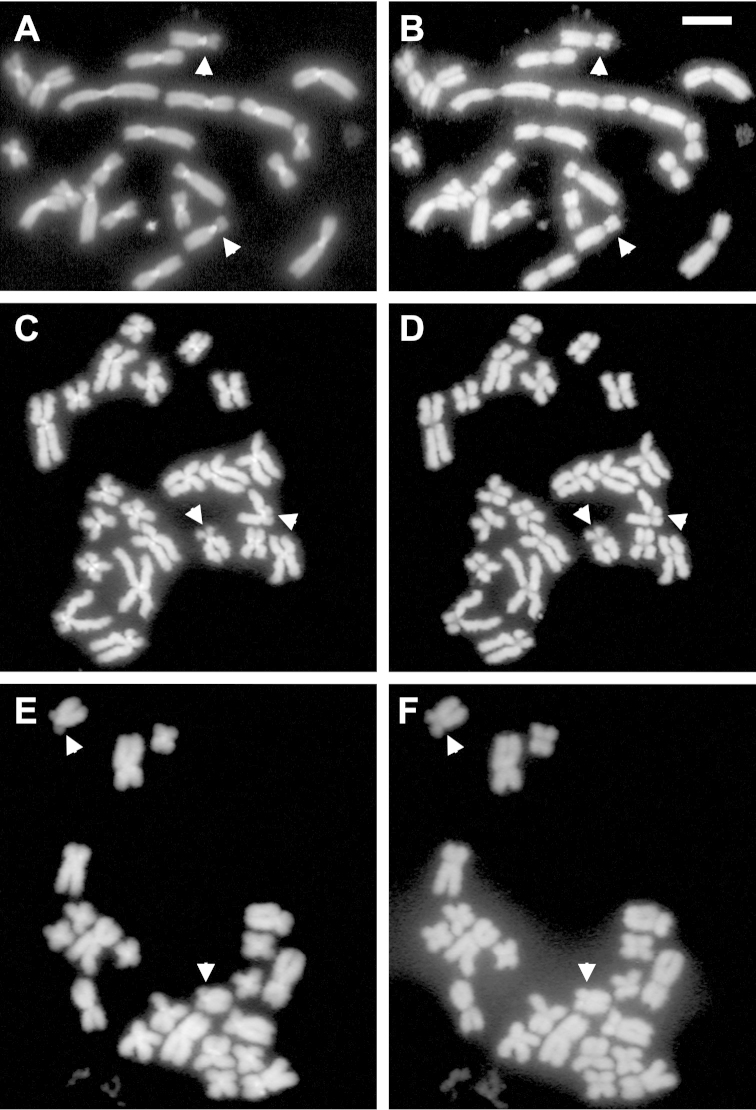
Mitotic metaphases of *Oreobates barituensis* (**A, B**), *Oreobates berdemenos* (**C, D**) and *Oreobates discoidalis* (**E, F**) stained with DAPI (**A, C, E**) and CMA_3_ (**B, D, F**), arrowheads point pairs 7. Bar = 10 µm.

**Figure 3.**
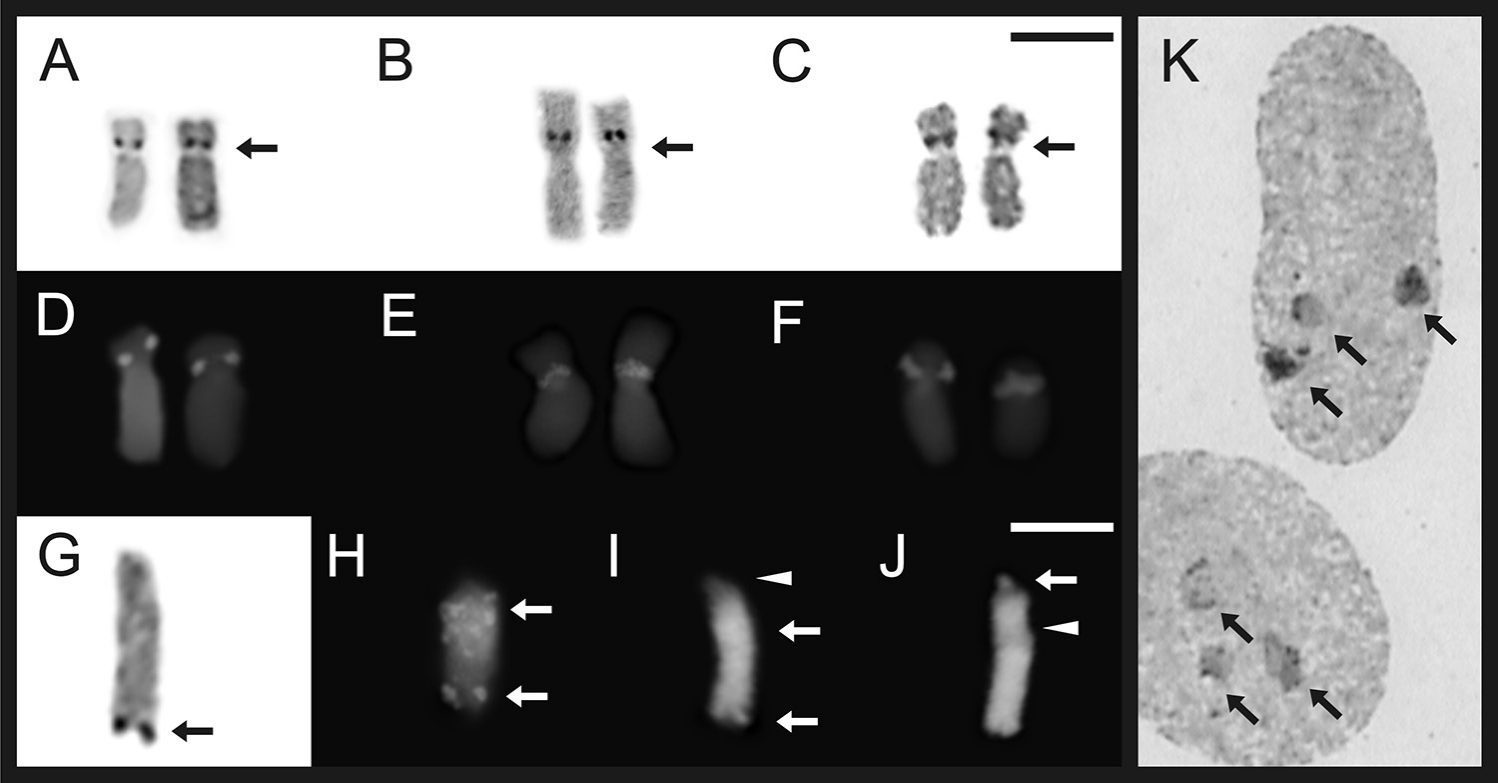
NORs bearing chromosome pairs (**A–C**) and rDNA (**D–F**), in *Oreobates barituensis* (**A, D**), *Oreobates berdemenos* (**B, E**) and *Oreobates discoidalis* (**C, F**). B_T_ chromosome in *Oreobates barituensis* showing positive NORs (**G**), active and inactive rDNA (**H**), CMA_3_ (**I**) and DAPI (**J**). Two interphase cells with three active NORs after silver staining (**K**). Black arrows indicate Ag-NORs, white arrows and arrowheads shows positive and negative fluorescent marks, respectively. Bars = 10 µm.

*Oreobates barituensis*, *Oreobates berdemenos*, and *Oreobates discoidalis* shared diploid karyotypes with 22 bi-armed chromosomes (2n = 22; FN = 44). Pairs 1, 2, 5, 6, 8–11 were metacentric, while 3, 4 and 7 submetacentric (Fig. 1; Table 1). In all specimens analyzed of the three species, the C-banding technique showed a high predominance of centromeric and pericentromeric heterochromatin, as well as an interstitial band on the long arm pairs 6 (Fig. 1D–F). However, this band varied in its staining intensity or even was absent in some metaphases of a given slide. DAPI/CMA_3_ fluorochromes staining, evidence DAPI positive marks at centromeric and pericentromeric regions on almost all chromosomes (CMA_3_ negative). In the three species, CMA_3_ positive marks (DAPI negative) were evident only on pericentromeric position over pair 7, coincident with secondary constriction sites (Fig. 2), Ag-NORs (Fig. 3A–C) and with the hybridization signals of 18S rDNA probe after FISH experiments (Fig. 3D–F).

**Table 1.** Chromosome morphology in the three species of *Oreobates*. Chromosome types according to Green and Sessions (1991), Centromeric Index (CI), metacentric (m: 0.500 to 0.375); submetacentric (sm: 0.374 to 0.250); SD = Standard Deviation.

Chromosome number	1	2	3	4	5	6	7	8	9	10	11
***Oreobates barituensis***											
%Set	15%	12%	11%	11%	10%	9%	8%	7%	6%	6%	6%
CI ± SD	0.43 ± 0.02	0.38 ± 0.03	0.37 ± 0.05	0.28 ± 0.06	0.45 ± 0.02	0.43 ± 0.03	0.34 ± 0.04	0.46 ± 0.02	0.46 ± 0.02	0.45 ± 0.03	0.47 ± 0.03
**Type**	**m**	**m**	**sm**	**sm**	**m**	**m**	**sm**	**m**	**m**	**m**	**m**
***Oreobates berdemenos***											
%Set	15%	12%	11%	11%	10%	9%	8%	7%	6%	6%	6%
CI ± SD	0.42 ± 0.02	0.38 ± 0.02	0.35 ± 0.05	0.25 ± 0.04	0.44 ± 0.02	0.45 ± 0.02	0.33 ± 0.04	0.45 ± 0.05	0.47 ± 0.02	0.45 ± 0.03	0.46 ± 0.03
**Type**	**m**	**m**	**sm**	**sm**	**m**	**m**	**sm**	**m**	**m**	**m**	**m**
***Oreobates discoidalis***											
%Set	15%	12%	11%	10%	10%	9%	8%	7%	6%	6%	6%
CI ± SD	0.44 ± 0.02	0.39 ± 0.02	0.37 ± 0.04	0.29 ± 0.04	0.45 ± 0.02	0.44 ± 0.03	0.27 ± 0.05	0.46 ± 0.02	0.46 ± 0.03	0.43 ± 0.05	0.48 ± 0.01
**Type**	**m**	**m**	**sm**	**sm**	**m**	**m**	**sm**	**m**	**m**	**m**	**m**

**Figure 4.**
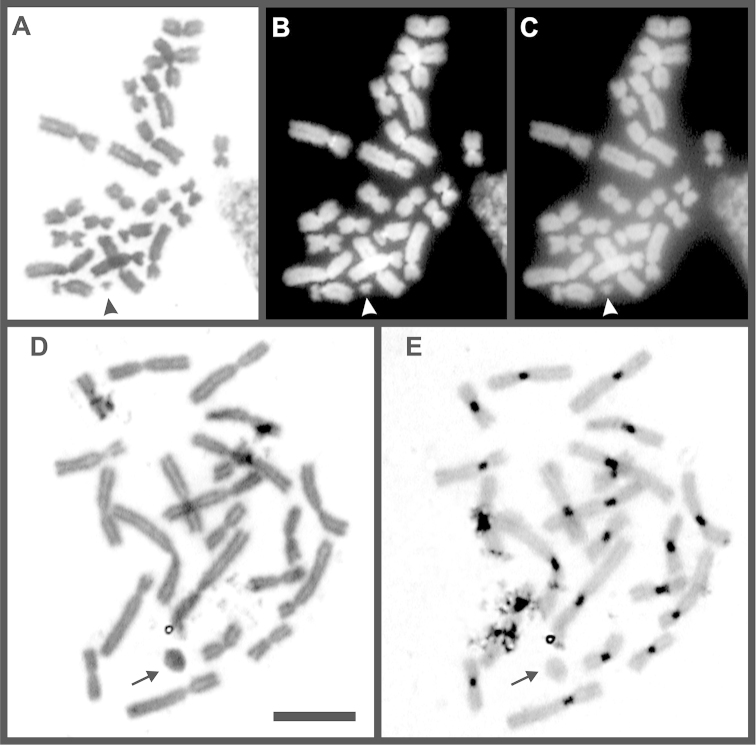
The small B_st_ chromosome (arrowhead) in *Oreobates barituensis* with conventional staining (**A**), DAPI (**B**) and CMA_3_ (**C**). B_d_ chromosome (arrow) in *Oreobates berdemenos* evidenced after Giemsa (**D**) and C-banding (**E**). Bar = 10 µm.

We found variations in the chromosome number in eight specimens of *Oreobates barituensis* as a consequence of two different B chromosomes (Fig. 1G–J; 3G–J; 4). Both supernumeraries were present in every somatic cell analyzed (mitotically stable). One of them, was a telocentric large-sized chromosome, arbitrarily named herein B_T_, which reached a maximum of one per cell (2n = 22 + B_T_). It was observed in three specimens from Peña Alta (LGE 6203, 4784–5) and two from Normenta (MSA 176, 180). C–banding revealed that this chromosome has a significant amount of heterochromatin in the whole arm, mainly visible in the centromeric region (Fig. 1I). The heterochromatin was DAPI positive/CMA_3_ negative with an interstitial mark DAPI negative/CMA_3_ positive (Fig. 3I, J). The B_T_ chromosome showed terminal active Ag-NOR sites after silver staining (Fig. 3G). In situ hybridization with the ribosomal 18S probe showed a terminal mark on B_T_ matching with Ag-NORs, but also brightly interstitial rDNA heterochromatin associated with negative Ag-NORs (Fig. 3H). The other supernumerary was a subtelocentric small chromosome, named as B_st_, found in the three specimens analyzed from Tiraxi (MSA 168, 195; LGE 9652). While a single individual carried only one B (2n = 22 + B_st_), the others brought it in a double dose (2n = 22 + 2B_st_). After C-banding, this small sized B_st_ stained darker than A chromosomes, with a conspicuous darker heterochromatic centromere (Fig. 1J), positive for DAPI (Fig. 4A–C).

In addition, eight specimens of *Oreobates barituensis* (LGE 4785; 6202; MSA 127–8, 161, 164, 174, 177), four *Oreobates berdemenos* (FML 24626, MSA 138, 142–3), and one *Oreobates discoidalis* (FML 24513), showed variations in the chromosome number attributable to dot-shaped and mitotically unstable supernumerary chromosomes, named here B_d_. These elements share a similar shape with no evident primary constrictions, and no more than one per metaphase was observed (i.e. 2n = 22; 22 + B_d_), with an occurrence below 50% per individual. C-banding and DAPI staining showed mostly a euchromatic nature of these elements (Fig. 4E).

**Figure 5.**
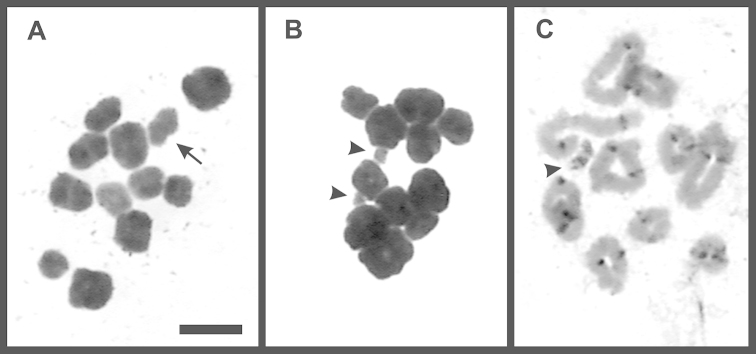
Meiosis I in specimens of *Oreobates barituensis*. Metaphase I with conventional staining showing Bs as univalent: one B_T_ (**A**) and two B_st_ with negative pyknosis (**B**). Diakinesis after C- banding with a bivalent B_st_ (**C**). Arrow and arrowheads indicate the B_T_ and B_st_ respectively.

The Meiosis I analyses on males of *Oreobates barituensis* with supernumerary chromosomes B_T_ and B_st_ evidenced the presence of 11 bivalents, corresponding to the A standard complement in addition to Bs. The B_T_ chromosome occurred as a single univalent with not differentiable pyknosis from other chromosomes (Fig. 5A). On the other hand, metaphases I from specimens with B_st_ showed a clearer staining than As (Fig. 5B). These elements were always observed as an univalent, and in those individuals carrying two Bs, both were detected as univalents (Fig. 5B) or even paired as a bivalent (Fig. 5C). In the latter case, the association between both B_st_ was euchromatic rather than heterochromatic (Fig. 5C). Meiosis of these elements from individuals carrying B_d_ supernumeraries could not be studied due to poor quality of preparations.

## Discussion

The genus *Oreobates* is composed of 23 species (Frost 2015), of which four of them were cytogenetically studied: *Oreobates barituensis* and *Oreobates berdemenos* (this work), *Oreobates discoidalis* (Brum-Zorrilla and Sáez 1968, Schmid 2010, this work), and *Oreobates crepitans* (Siqueira et al. 2009). *Oreobates* species share a similar morphology of chromosomes, as well as C-banding patterns and the location of NORs. The interstitial heterochromatic band observed in the long arm of pair 6 on specimens of the three species of *Oreobates* studied here, was not previously reported for *Oreobates crepitans* by Siqueira et al. (2009). However, with the use of similar procedures than these authors for the C-banding protocol, the band was not detectable or was variably marked; suggesting that condensation of chromosomes may play an important role in its detection.

Within the subfamily Holoadeninae, the chromosomes of only 8 of 119 recognized species were studied (Schmid et al. 2010, and references therein). The 2n = 22 with all biarmed chromosome pairs (FN = 44) shared by *Oreobates* species are also present in *Phrynopus barthlenae* Lehr & Aguilar, 2002. However, *Barycholos ternetzi* (Miranda-Ribeiro, 1937) shows a 2n = 22; FN = 38, *Euparkerella brasiliensis* (Parker, 1926) 2n = 20; FN = 40, and *Holoaden bradei* Lutz, 1958 2n = 18; FN = 36 (Schmid et al. 2010). Although, *Lynchius* Hedges, Duellman & Heinicke, 2008, *Oreobates* and *Phrynopus* Peters, 1873, were recurrently recovered as related groups in several phylogenetic hypotheses (Hedges et al. 2008, Pyron and Wiens 2010, Padial et al. 2014), the scarcity of data do not allow a clear understanding of chromosome character distribution among Holoadeninae (complete absence of data for *Bryophryne*, *Hypodactylus*, *Niceforonia*, *Noblella*, and *Psychrophrynella*). However, the available karyological information suggests that a whole-biarmed karyotype of 22 chromosomes (FN = 44) is shared by species of the clade comprising *Lynchius*, *Oreobates*, and *Phrynopus*.

B chromosomes are widespread among eukaryotes (Beukeboom 1994, Camacho 2005), and to date have been formally reported in 20 anuran species (Green 2004 and references therein, Lanzone et al. 2008, Schmid et al. 2010 and references therein, Milani et al. 2011, Hernández-Guzmán et al. 2011, Suárez et al. 2013, Mezzasalma et al. 2015). Schmid et al. (2010) analyzed with conventional solid staining the chromosomes of 11 specimens of *Oreobates discoidalis*, collected by J.P. Bogart in 1969 in northwestern Argentina (Aguas Negras, Jujuy). These authors found two different telocentric B chromosomes, both showing a mosaic in the chromosome number between cells of a same specimen (mitotically unstable), occurring at most once per cell. One of those Bs was smaller, similar in size to the pair 11 of the A complement, whereas the other one was larger than pair 5, resembling the B_T_ described in the present work for *Oreobates barituensis*. As the specimens studied by Schmid et al (2010) were collected in late 1960s, with *Oreobates discoidalis* (= *Eleutherodactylus discoidalis*) being then the unique species recognized in Argentina, we cannot ascertain whether B_T_ chromosome were present in *Oreobates barituensis* (this paper) and *Oreobates discoidalis* (Schmid et al. 2010) or only in *Oreobates barituensis*. However, an intriguing question is the lack of mitotic instability observed by us on this element as compared with previous reports. At present, the most accepted models for long-term evolution of B chromosomes states that the cytological behavior of Bs polymorphisms can change over time. A selfish (or parasitic) B may develop into neutral (or near-neutral) through a stabilization process induced by the standard genome of the host species (Camacho et al. 1997, Zurita et al. 1998, Camacho et al. 2000), or alternatively by regularizing their own pairing during meiosis (Araújo et al. 2001, 2002). While the mitotic instability of B chromosomes can be interpreted as a mechanism for accumulation, this phenomenon hinders an organism (or a population) to fit to an optimal number of these elements, and unless it favors the transmission of Bs on germinal cells (i.e. by premeiotic accumulation) it would play a negative role in their long-term persistence (Nur 1963, 1969). Cavallaro et al. (2000) demonstrated that mitotic instability may take part as a possible mechanism for Bs to increase their frequency and thus invade populations. The authors observed over a ten year period a significant rise in the frequency of a B chromosome in the fish *Prochilodus lineatus* (Valenciennes, 1837). This was correlated with a decrease in the mitotic instability of the chromosome (i.e. Mitotic stabilization), suggesting that the population studied was likely under the last phase of B chromosome invasion. A possible explanation for the discrepancies observed between the present work and that of Schmid et al. (2010) concerning the mitotic behavior of the B_T_ in *Oreobates barituensis* is that the instability of this element was abolished over time in the studied population (almost 50 years).

NOR-bearing B chromosomes were reported in 27 species of plants and 25 of animals [three of them anurans: *Eleutherodactylus gundlachi*, *Gastrotheca espeletia* Duellman & Hillis, 1987, and *Spea hammondii* (Baird, 1859)], showed to be supernumerary chromosomes carrying rDNA detected by silver staining and/or FISH with a rDNA probe (Green 1990, Jones 1995, Jones and Díez 2004, Silva and Yonenaga- Yassuda 2004, Camacho 2005, Cabrero and Camacho 2008, Acosta and Moscone 2010, Schmid et al. 2010, Ruiz-Estévez et al. 2013, Silva et al. 2014). The B_T_ chromosome in *Oreobates barituensis* displayed active NORs in mitoses as well as in interphase nuclei, but also has interstitial heterochromatin associated with inactive 18S rDNA. A tempting target for further studies to test the origin of this B chromosome are the pair of A chromosomes carrying NORs (pair 7). It must be considered that B chromosomes may suffer a further degeneration after their origin, thus becoming heterochromatic and loosing homology with their precursors (Green 1990, Camacho et al. 2000), in addition to the observed mobility nature of rDNA sequences by transposition between non-homologous chromosomes. These facts prevent us from hypothesizing about the possible origin of this element, as B chromosomes would have acquired rDNA subsequent to their formation (Houben et al. 2013, and references therein).

Two other types of supernumerary chromosomes observed in *Oreobates barituensis* were a small subtelocentric and heterochromatic B_st_ that occurs in a high prevalence in the locality of Tiraxi, and the euchromatic dot-like B_d_, which is mitotically unstable. Interestingly, specimens of the two other species of *Oreobates* analyzed herein also showed supernumeraries B_d_ of similar size, and smaller than the smallest pair of the A complement. B chromosomes, which lack a functional centromere would be lost by drift (Camacho et al. 1997). The low transmission efficiency of these elements observed in somatic cells (lower than 50% per individual) due to mitotic instability would impede them to survive as true B chromosomes, unless their transmission ability would be increased in the gametes by acquiring a functional centromere. It is remarkable that the other small supernumerary elements described as B_st_ differed from those B_d_ by the presence of a conspicuous centromere, and because they were recorded in almost all somatic and germinal cells.

Finally, the available cytogenetic data points to the Brachycephaloidea as an extremely diverse group, with 2n ranging from 16 to 38, and FN = 26–52 (De Weese 1975, Bogart 1991, Bogart and Hedges 1995, Campos and Kasahara 2006, Green and Sessions 2007, Schmid et al. 2010). However, the occurrence of species bearing B chromosomes is not higher than in other anurans groups. Under this scenario, Schmid et al. (2010, 2012) reported the occurrence of “spontaneous somatic supernumerary marker chromosomes” as a common feature observed amongst Brachycephaloidea, at first described in the discoglossid frog *Hoplobatrachus tigerinus* (Daudin, 1802) by Yadav (1973). These elements are considered to be originated as a consequence of spontaneous chromosomal rearrangements. Like B chromosomes, are variable in composition and structure of chromatin, morphology, and behavior during mitosis, denoting thus an heterogeneous chromosome type found in more than 50 species of brachycephaloids frogs (Schmid et al. 2010, 2012). This fact leads to the issue that there might be B chromosomes or nascent Bs undetected among them, pointing to Brachycephaloidea as an interesting group for studying the origin and evolution of B chromosomes in Anurans.

## Supplementary materials

Supplementary material 1 Specimens of *Oreobates* examined Data type: specimens data This dataset is made available under the Open Database License (http://opendatacommons.org/licenses/odbl/1.0/). The Open Database License (ODbL) is a license agreement intended to allow users to freely share, modify, and use this Dataset while maintaining this same freedom for others, provided that the original source and author(s) are credited.Juan Martín Ferro, Alberto Taffarel, Darío Cardozo, Jimena Grosso, María Pía Puig, Pablo Suárez, Mauricio Sebastián Akmentins, Diego Baldo

## References

[B1] Acosta MC Moscone E (2011) B chromosomes in *Nierembergia aristata* (Solanaceae): nucleolar activity and competition with the A chromosomes. Cytogenetic and Genome Research 132: 105–12. doi: 10.1159/000320705 20924164 10.1159/000320705

[B2] Akmentins MS (2011) Vocal repertoire of two species of *Oreobates* Jiménez de la Espada, 1872 (Anura: Strabomantidae) of the Yungas Andean Forest, NW Argentina. Journal of Natural History 45: 1789–1799. doi: 10.1080/00222933.2011.560967

[B3] Araújo SMSR Pompolo SG Perfectti F Camacho JPM (2002) Integration of a B chromosome into the A genome of a wasp. Procedings of the Royal Society B: Biological Sciences 269: 1475–1478. doi: 10.1098/rspb.2001.1613 10.1098/rspb.2002.2040 PMC1691057 12137577

[B4] Araújo SMSR Pompolo SG Perfectti F Camacho J (2002) Integration of a B chromosome into the A genome of a wasp, revisited. Procedings of the Royal Society B: Biological Sciences 269: 1475–1478. doi: 10.1098/rspb.2002.2040 10.1098/rspb.2002.2040 PMC1691057 12137577

[B5] Beukeboom LW (1994) Bewildering Bs: an impression of the 1^st^ B-Chromosome Conference. Heredity 73: 328–336. doi: 10.1038/hdy.1994.140

[B6] Bogart J (1991) The Influence of Life History on Karyotypic Evolution in Frogs. In: Green DM Sessions SK (Eds) Amphibian Cytogenetics and Evolution. New York, Academic Press, San Diego, 233–258. doi: 10.1016/b978-0-12-297880-7.50015-9

[B7] Bogart J Hedges S (1995) Rapid chromosome evolution in Jamaican frogs of the genus *Eleutherodactylus* (Leptodactylidae). Journal of Zoology 235: 9–31. doi: 10.1111/j.1469-7998.1995.tb05124.x

[B8] Brum-Zorrilla N Sáez FA (1968) Chromosomes of Leptodactylidae (Amphibia Anura). Experientia 24: 969. doi: 10.1007/bf02138689 10.1007/BF02138689 5709058

[B9] Cabrero J Camacho J (2008) Location and expression of ribosomal RNA genes in grasshoppers: Abundance of silent and cryptic loci. Chromosome Research 16: 595–607. doi: 10.1007/s10577-008-1214-x 18431681 10.1007/s10577-008-1214-x

[B10] Camacho JPM (2005) B chromosomes. In: Gregory TR (Ed.) Evolution of the Genome. Academic Press, 223–286. doi: 10.1016/B978-012301463-4/50006-1

[B11] Camacho J Shaw MW López-León MD López-León MD Pardo MC Cabrero J (1997) Population dynamics of a selfish B chromosome neutralized by the standard genome in the grasshopper *Eyprepocnemis plorans*. The American Naturalist 149: 1030–1050. doi: 10.1086/286037 10.1086/286037 18811262

[B12] Camacho JPM Sharbel TF Beukeboom LW (2000) B-chromosome evolution. Philosophical Transactions of the Royal Society B: Biological Sciences 355: 163–78. doi: 10.1098/rstb.2000.0556 10.1098/rstb.2000.0556 PMC1692730 10724453

[B13] Campos JRC Kasahara S (2006) Os cromossomos dos anfíbios anuros do gênero *Eleutherodactylus* (Anura: Leptodactylidae: Eleutherodactylinae). Publicatio UEPG: Ciências Biológicas e da Saúde, Ponta Grossa 12: 27–38. http://www.revistas2.uepg.br/index.php/biologica/article/viewArticle/426

[B14] Cavallaro ZI Bertollo LAC Perfectti F Camacho J (2000) Frequency increase and mitotic stabilization of a B chromosome in the fish *Prochilodus lineatus*. Chromosome Research 8: 627–634. doi: 10.1023/A:1009242209375 11117359 10.1023/a:1009242209375

[B15] Díaz L Hedges S Schmid M (2012) A new cryptic species of the genus *Eleutherodactylus* (Amphibia: Anura: Eleutherodactylidae) from Cuba. Zootaxa 3220: 44–60. http://www.mapress.com/zootaxa/2012/f/z03220p060f.pdf

[B16] De Weese J (1975) Chromosomes in *Eleutherodactylus* (Anura: Leptodactylidae). Mammalian Chromosome Newsletter 16: 121–123.

[B17] Ferrari L Vaira M (2008) Descripción del canto de anuncio de una población Argentina de *Oreobates discoidalis* (Anura: Strabomantidae). Cuadernos de Herpetología 22: 81–85.

[B18] Frost DR (2015) Amphibian species of the world: an online reference. Version 6.0. Electronic Database accessible at http://research.amnh.org/herpetology/amphibia/index.html. American Museum of Natural History, New York, USA.

[B19] Green DM Sessions SK (1991) Nomenclature for chromosomes. In: Green DM Sessions SK (Eds) Amphibian Cytogenetics and Evolution. New York, Academic Press, San Diego, 431–432. doi: 10.1016/b978-0-12-297880-7.50021-4

[B20] Green DM Sessions SK (2007) Karyology and Cytogenetics. In: Heatwole H Tyler M (Eds) Amphibian Biology Surrey Beatty and Sons, Chipping Norton, vol. 7, 2756–2841.

[B21] Green D (1990) Muller’s Ratchet and the evolution of supernumerary chromosomes. Genome 33: 818–824. doi: 10.1139/g90-123

[B22] Green DM (2004) Structure and evolution of B chromosomes in amphibians. Cytogenetic and Genome Research 106: 235–242. doi: 10.1159/000079293 15292597 10.1159/000079293

[B23] Hedges S Duellman W Heinicke M (2008) New World direct-developing frogs (Anura: Terrarana): molecular phylogeny, classification, biogeography, and conservation. Zootaxa 1737: 1–182. http://datadryad.org/handle/10255/dryad.7143

[B24] Hernández-Guzmán J Arias-Rodriguez L Indy JR (2011) Los cromosomas meióticos de la rana arborícola *Smilisca baudinii* (Anura: Hylidae). Revista de Biología Tropical 59: 355–362. doi: 10.15517/rbt.v59i1.3204 21516655

[B25] Houben A Banaei-Moghaddam AM Klemme S (2013) Biology and evolution of B chromosomes. In: Leitch IJ (Ed.) Plant Genome Diversity Volume 2 Springer, Berlin, Germany, 149–165. doi: 10.1007/978-3-7091-1160-4_10

[B26] Howell WM Black DA (1980) Controlled silver–staining of nucleolus organizer regions with a protective colloidal developer: a 1–step method. Experientia 36: 1014–1015. doi: 10.1007/BF01953855 6160049 10.1007/BF01953855

[B27] Jones RN (1995) Tansley review no. 85: B chromosomes in plants. New Phytologist 131: 411–434. doi: 10.1111/j.1469-8137.1995.tb03079.x 10.1111/j.1469-8137.1995.tb03079.x 33863119

[B28] Jones RN Diez M (2004) The B chromosome database. In: B chromosomes in the eukaryote genome. Cytogenetic and Genome Research 106: 149–150. doi: 10.1159/000079280 15292584 10.1159/000079280

[B29] Jones RN Rees H (1982) B chromosomes. Academic Press, 1–266.

[B30] Kaiser H Barrio-Amorós CL Rivas GA Steinlein C Schmid M (2015) Five new species of *Pristimantis* (Anura: Strabomantidae) from the coastal cloud forest of the Península de Paria, Venezuela. Journal of Threatened Taxa 7(4): 7047–7088. doi: 10.11609/JoTT.o4197.7047-88

[B31] Lanzone C Baldo D Rosset SD (2008) Meiotic differentiation in two allopatric population groups of the tetraploid frog *Odontophrynus americanus* from Argentina. Herpetological Journal 18: 213–222. http://www.ingentaconnect.com/content/bhs/thj/2008/00000018/00000004/art00005

[B32] Mezzasalma M Gaetano FG Petraccioli OA Guarino FM (2015) Chromosome analyses of *Pseudhymenochirus merlini* and *Hymenochirus boettgeri* provide new insights into the chromosome evolution in the anuran family Pipidae. Zoologischer Anzeiger 258: 47–53. doi: 10.1016/j.jcz.2015.07.001

[B33] Milani M Cassini CS Recco-Pimentel SM Lourenço LB (2011) Karyotypic data detect interpopulational variation in *Physalaemus olfersii* and the first case of supernumerary chromosome in the genus. Animal Biology Journal 2: 21–28.

[B34] Nur U (1963) A mitotically unstable supernumerary chromosome with an accumulation mechanism in a grasshopper. Chromosoma 14: 407–422. doi: 10.1007/BF00326786 13939243 10.1007/BF00326786

[B35] Nur U (1969) Mitotic instability leading to an accumulation of B-chromosomes in grasshoppers. Chromosoma 27: 1–19. doi: 10.1007/BF00326108 5820707 10.1007/BF00326108

[B36] Padial JM Grant T Frost DR (2014) Molecular systematics of terraranas (Anura: Brachycephaloidea) with an assessment of the effects of alignment and optimality criteria. Zootaxa 3825: 1–132. doi: 10.11646/zootaxa.3825.1.1 24989881 10.11646/zootaxa.3825.1.1

[B37] Pereyra MO Cardozo DE Baldo J Baldo D (2014) A new species of *Oreobates* (Anura, Craugastoridae) from Northeastern Argentina. Herpetologica 70: 211–227. doi: 10.1655/HERPETOLOGICA-D-13-00072

[B38] Pinkel D Straume T Gray JW (1986) Cytogenetic analysis using quantitative, high sensitivity, fluorescence hybridization. Proceedings of the National Academy of Sciences of the United States of America 83: 2934–2938. doi: 10.1073/pnas.83.9.2934 3458254 10.1073/pnas.83.9.2934 PMC323421

[B39] Pyron RA Wiens JJ (2011) A large-scale phylogeny of Amphibia including over 2800 species, and a revised classification of extant frogs, salamanders, and caecilians. Molecular Phylogenetics and Evolution 61: 543–83. doi: 10.1016/j.ympev.2011.06.012 21723399 10.1016/j.ympev.2011.06.012

[B40] Ruíz-Estévez M López-León MD Cabrero J Camacho JPM (2013) Ribosomal DNA is active in different B chromosome variants of the grasshopper *Eyprepocnemis plorans*. Genetica 141: 337–345. doi: 10.1007/s10709-013-9733-6 24008810 10.1007/s10709-013-9733-6

[B41] Reeves A Tear J (2000) Micromeasure version 3.3. http://www.colostate.edu/Depts/Biology/MicroMeasure

[B42] Schmid M Steinlein C Bogart JP Feichtinger W León P La Marca E Díaz LM Sans A Chen SH Hedges SB (2010) The chromosomes of terraranan frogs. Insights into vertebrate cytogenetics. Cytogenetic and Genome Research 130-131: 1–568. doi: 10.1159/000301339 21063086 10.1159/000301339

[B43] Schmid M Steinlein C Bogart JP Feichtinger W Haaf T Nanda I del Pino EM Duellman WE Hedges SB (2012) The hemiphractid frogs. Phylogeny, embryology, life history, and cytogenetics. Cytogenetic and Genome Research 138: 69–384. doi: 10.1159/000343460 23429349 10.1159/000343460

[B44] Sumner AT (1972) A simple technique for demonstrating centromeric heterochromatin. Preliminary notes. Experimental Cell Research 75: 304–306 4117921 10.1016/0014-4827(72)90558-7

[B45] Schweizer D (1976) Reverse fluorescent chromosome banding with chromomycin and DAPI. Chromosoma 58: 307–324. doi: 10.1007/BF00292840 137107 10.1007/BF00292840

[B46] Siqueira S Aguiar Jr O Pansonato A Giaretta AA Strüssmann C Martins I Recco-Pimentel S (2009) The karyotype of three Brazilian Terrarana frogs (Amphibia, Anura) with evidence of a new *Barycholos* species. Genetics and Molecular Biology 32: 470–476. doi: 10.1590/S1415-47572009005000044 21637508 10.1590/S1415-47572009005000044 PMC3036057

[B47] Silva MJ Yonenaga-Yassuda Y (2004) B chromosomes in Brazilian rodents. Cytogenetic and Genome Research 106: 257–263. doi: 10.1159/000079296 15292600 10.1159/000079296

[B48] Silva DMZ Pansonato-Alves JC Utsunomia R Araya-Jaime C Ruiz-Ruano F Natal Daniel S Huashimoto DT Oliveira C Camacho JPM Porto-Foresti F Foresti F (2014) Delimiting the origin of a B chromosome by FISH mapping, chromosome painting and DNA sequence analysis in *Astyanax paranae* (Teleostei, Characiformes). PLoS ONE 9: . doi: 10.1371/journal.pone.0094896 10.1371/journal.pone.0094896 PMC3988084 24736529

[B49] Suárez P Cardozo D Baldo D Pereyra MO Faivovich J Orrico VGD Catroli GF Grabiele M Bernarde PS Nagamachi CY Haddad CB Pieczarka JC (2013) Chromosome evolution in dendropsophini (Amphibia, Anura, Hylinae). Cytogenetic and Genome Research 141: 295–308. doi: 10.1159/000354997 24107475 10.1159/000354997

[B50] Vaira M Ferrari L (2008) A new species of *Oreobates* (Anura : Strabomantidae) from the Andes of northern Argentina. Zootaxa 50: 41–50. http://www.mapress.com/zootaxa/2008/f/z01909p036f.pdf

[B51] Yadav J (1974) Spontaneous occurrence of aneuploidy in somatic cells of Rana tigrina (Ranidae: Anura). Experientia 30: 1070–1072. doi: 10.1007/BF01939016 4547306 10.1007/BF01939016

[B52] Zurita S Cabrero J López-León MD Camacho JPM (1998) Polymorphism regeneration for a neutralized selfish B chromosome. Evolution 52: 274–277. doi: 10.2307/2410945 10.1111/j.1558-5646.1998.tb05163.x 28568137

