# Supplementary material for: Cytogenetic characterization and B chromosome diversity in direct-developing frogs of the genus Oreobates (Brachycephaloidea, Craugastoridae)
Source: Comp Cytogenet. 2016 Mar 21;10(1):141–56. doi: 10.3897/CompCytogen.v10i1.5718 (PMC4856932; doi:10.3897/CompCytogen.v10i1.5718)
Supplement: Supplementary material 1 — Specimens of Oreobates examined [file CompCytogen-010-141-s001.pdf]

Supplementary File S1. Specimens of *Oreobates* examined.

*Oreobates barituensis*—ARGENTINA. Jujuy: Departamento Ledesma: Calilegua, LGE 6195-6200 (four males, one female, and one juvenile); Normenta, LGE 9457–8, 9460–1, MSA 175–7, 179–80 (six males, two females, and one juvenile); Mesada de las Colmenas, LGE 4769 (one female). Departamento Valle Grande: San Francisco, LGE 6201–3, LGE 4784-6 (four males and two juveniles). Departamento Santa Bárbara: El Fuerte, MSA 127–8, LGE 9452–4, 9462 (five males and one female). Salta: Departamento Santa Victoria: El Azaray, LGE 9447–8, 9450–1, MSA 160–4 (nine males). Departamento General Manuel Belgrano: Tiraxi, LGE 9455, 9652, MSA 195 (three males).

*Oreobates berdemenos*—ARGENTNA. Jujuy: Departamento Ledesma: Abra Colorada, FML 24622, 24626, LGE 2530, 8414, 8433–4 (3 males and 3 females). Salta: Departamento Santa Victoria: Reserva Nogalar de los Toldos, MSA 138–44, 148 (eight males).

*Oreobates discoidalis*—ARGENTINA. Tucumán: Departamento Lules: Villa Nougues, FML 24505, 24507, 24509–10, 24512–3, LGE 2539, LGE 7422–4 (four males and six females).
